# Attention deficit hyperactivity disorder in adults who present with self-harm: a comparative 6-month follow-up study

**DOI:** 10.1186/s12888-022-04057-0

**Published:** 2022-06-24

**Authors:** Petter Olsson, Stefan Wiktorsson, Lotta M. J. Strömsten, Ellinor Salander Renberg, Bo Runeson, Margda Waern

**Affiliations:** 1 Department of Psychiatry and Neurochemistry, University of Gothenburg, Blå stråket 15, vån 3, SU/S, 413 45 Göteborg, Sweden; 2 Region Västra Götaland, Sahlgrenska University Hospital, Psychosis Clinic, Mölndal, Sweden; 3 Department of Psychology, Umeå University, Umeå, Sweden; 4 Department of Clinical Sciences, Psychiatry, Umeå University, Umeå, Sweden; 5 Department of Clinical Neuroscience, Centre for Psychiatry Research, Karolinska Institute and Stockholm Health Care Services, Region Stockholm, Stockholm, Sweden

**Keywords:** ADHD, Suicide, Attempt, Adults

## Abstract

**Background:**

ADHD is common in psychiatric populations. This study aimed to compare clinical characteristics in adults with and without ADHD who presented with self-harm, and to compare later risk of suicidal behaviour within 6 months.

**Methods:**

Eight hundred four adults presented with self-harm (with and without suicidal intent) at psychiatric emergency services at three Swedish hospitals. Persons with a discharge ICD-10 diagnosis F90.0-F90.9 or a prescription for ADHD medication were considered to have ADHD (*n* = 93). Medical records were reviewed for evidence of subsequent suicide attempts (SA) within 6 months; suicides were identified by national register.

**Results:**

Recent relationship problems were more prevalent in the ADHD group. While the index episodes of those with ADHD were more often non-suicidal, and actual SAs more often rated as impulsive, medical lethality at presentation did not differ in attempters with and without ADHD. Subsequent SAs (fatal or non-fatal) were observed in 29% of the ADHD group and 20% in all others (*P =* .005). A logistic regression model showed elevated risk of suicidal behaviour during follow-up in the ADHD group (OR = 1.70, CI 1.05–2.76), although a final regression model suggested that this association was partly explained by age and comorbid emotionally unstable personality disorder.

**Conclusions:**

Findings highlight the need for clinicians to take self-harm seriously in adults with ADHD.

**Supplementary Information:**

The online version contains supplementary material available at 10.1186/s12888-022-04057-0.

## Background

Attention-deficit/hyperactivity disorders (ADHD) are common in psychiatric populations, and there are indicators that suicide risk may be heightened in persons with these disorders. In children and adolescents, studies have shown that ADHD predicts suicidal behaviour [1–5]. This may in part be explained by the fact that children with ADHD are at increased risk of developing psychiatric comorbidities predisposing for suicide, including depression, drug misuse and antisocial behaviour compared to their peers [6, 7]. Increased impulsivity and aggression have also been suggested to mediate the relationship between ADHD and suicidal behaviour [8], and to be associated with a history of suicide attempts [9].

Problems of hyperactivity and attention deficit and related comorbidities may persist into adulthood [10, 11]. Recent studies in young adult women with childhood ADHD showed that impairments are likely to remain in early adulthood [12] and predispose to suicide attempts as well as non-suicidal self-harm [13]. In males, an extensive review of longitudinal data showed an association between ADHD and completed suicide [14]. The authors of that review suggested that the heightened risk may in large part be attributed to an increased burden of comorbidities such as conduct disorders and depression. A Swedish register-based study showed an association between ADHD and increased risk of suicide and suicide attempts in both males and females aged 12–40 years [15]. Risk of suicidal behaviour remained elevated after adjustment for comorbid psychiatric disorders in that study, and a shared genetic risk for both ADHD and suicidal behaviour was demonstrated. While the above findings indicate several mechanisms by which hyperactivity disorders may be connected to suicidality in adults, prospective clinical studies are needed to inform clinicians of potential risk indicators in patient populations. The aims were to compare characteristics of patients with and without ADHD in a clinical cohort of adults who presented at psychiatric emergency services with self-harm regardless of intent, and to examine the prospective association between ADHD and short-term risk of suicidal behaviour.

## Methods

### Participants

In total 804 patients (541 women, 263 men) aged 18 and older were recruited and interviewed at three university hospitals (Sahlgrenska University Hospital in Gothenburg (*n* = 190), St Göran’s Hospital, Karolinska Institute, Stockholm (*n* = 479) and the Norrland University Hospital in Umeå (*n* = 135) in connection with a suicide attempt or an episode of non-suicidal self-injury (NSSI). As previously described [16], recruitments were carried out between April 2012 and March 2016. For participation, a patient had to be registered as a resident of the catchment area for one of the three hospitals. No specific diagnoses resulted in exclusion. However, if the attending physician determined that the patient would be unable to complete the interview due to insufficient language skills, cognitive ability, severe psychosis, delirium, dementia or severe somatic conditions, they were excluded. As previously reported [16], participation rates were as follows: Gothenburg, 70%, Stockholm, 68% and Umeå, 82% (total participation rate 71%). The median age of participants was 33 years (25th percentile = 23, 75th percentile = 50, range 18–95). Women (Mdn = 30) were younger than men (Mdn = 38). At baseline, 666 participants (83% of the total sample) presented with an actual suicide attempt, defined in accordance with the Columbia Suicide Severity Rating Scale (C-SSRS) [17] as an act of physical self-injury with at least some degree of intent to die. In the remaining 138 participants (17%), the baseline self-injury was without intent to die and was thus defined as an NSSI.

### Procedure

Participants were interviewed within a mean of 3.9 (Mdn = 3) days after emergency psychiatric consultation. Interview duration averaged 1–2 hours. All interviews were conducted by mental health staff (psychiatrists, psychiatric nurses, psychologists and psychiatric trainees) specially trained in the application of the assessment instruments [16]. As previously reported [16, 18], interrater agreement was very good-excellent for the C-SSRS [17] and Suicide Intent Scale (SIS) [19]. It was also excellent for the Suicide Assessment Scale (SUAS) total score (intraclass correlation, ICC = 0.99, *P* < .001).

### Instruments

AUDIT (Alcohol Use Disorders Identification Test) [20] was used to assess alcohol use and alcohol-related problems. In accordance with WHO guidelines, the cut-off for hazardous alcohol use was set to 8 points for men aged < 65 and 7 points for women and men aged > 65 [21]. For those with an actual suicide attempt at baseline, medical lethality was rated on a scale of 0–5 according to the C-SSRS item 21a [17]. For the purpose of this study, we set a cut-off point of 3 points or above to define high lethality (moderately severe physical injury requiring hospital care probably in an intensive care unit). The impulsivity trait was rated by the interviewer on a scale of 0–4 according to the Suicide Assessment Scale (SUAS), a scale designed specifically to rate symptoms previously shown to be related to suicide, independent of diagnosis [22]. For the impulsivity variable we set a cut-off of 2 points or above to define high impulsivity (I often have difficulty controlling my impulsive needs and drives, with limited knowledge of the consequences). Impulsivity in connection with actual suicide attempt at index was rated on a scale of 0–2 according to the Suicide Intent Scale (SIS) [19] item 15 (degree of preparation). In this study, a score of 0 (no preparations, impulsive act) defined the attempt as impulsive. Depressive symptoms were self-rated using the Montgomery-Åsberg Depression Rating Scale (MADRS-S) [23] and anxiety symptoms were rated with the self-rating version of the Brief Scale for Anxiety (BSA) [24].

### Diagnoses

Diagnoses in connection with the index episode were retrieved from psychiatric records. Persons with discharge diagnoses F90.0-F90.9 according to the International Statistical Classification of Diseases and Related Health Problems (ICD-10) were considered to have ADHD. Specific diagnoses in this group include ADHD, attention deficit disorder (ADD), dysfunction of attention, motor control and perception (DAMP) and other specified conditions of hyperactivity. In accordance with the Swedish study of Ljung and colleagues [15], having a prescription for ADHD medication was considered a proxy marker for ADHD, and persons prescribed such treatment were also considered to have ADHD for the purpose of this study. Persons with discharge diagnoses F32.0-F32.9 and/or F33.0-F33.9 according to ICD-10 were considered to have depression.

### Outcomes

Actual suicide attempts in accordance with the C-SSRS [17] within 6 months after the index episode were recorded through review of in- and outpatient records. Suicide deaths were identified through the National Cause of Death Register, which contains information on all deaths of registered residents in Sweden. Since 1961, the register has had an estimated coverage rate of > 99% [25]. Patient medical records were linked to the register using their unique Swedish personal number.

### Statistics

*T*-tests were used to investigate group differences between patients with and without ADHD/subsequent attempts. Chi-square tests were used to compare proportions between the same groups. Mann-Whitney U Test was used for age group comparisons (18–44, 45–64, 65+). Logistic regression models were used to calculate odds ratios for new episodes of self-harm with death intent and analyses were then adjusted for depression, emotionally unstable personality disorder (EUPD), trait impulsivity, sex and age (continuous variable). Similar regression models were created with data stratified by age group. Normality was assessed via visual inspection of histograms for deviations from normal distribution. All *t*-tests were cross-checked with non-parametric equivalents and yielded similar results. Statistical analyses were performed using SPSS® version 24 for Windows.

Missing data was investigated using the SPSS Missing Value Analysis (MVA) module and for SUAS, BSA and MADRS tested for missingness mechanism, using Little’s Missing Completely at Random (MCAR) test. Missing data for assessment scales, identified as MCAR or missing at random (MAR) values were imputed by single imputation, using expectation maximum (EM) algorithm. For SUAS, data was treated as MAR instead of MCAR, Little’s MCAR test; *χ*^*2*^ (246, *n* = 804) = 1372, *P =* .01. For cases with partial missing data (*n* = 38), in total 5% of SUAS data, values were imputed. Where data was not imputed, cases with missing data were excluded from the current analysis. Details on other analyses where cases were excluded due to missing data are included in Tables 2 and 3.

## Results

### Index self-harm episode

A diagnosis of ADHD (ICD-10 F90.0-F90.9) was noted in the case record in connection with the index episode for 76 persons (10%). An additional 17 persons who did not have an F90.0-F90.9 diagnosis were prescribed pharmacological treatment for ADHD at the time of the index episode. Thus, for the purpose of this study, a total of 93 persons (12%) were considered to have ADHD. Methods employed at the index NSSI/SA are shown by ADHD status in Table 1.

**Table 1:** Methods employed in connection with index episode of self-harm in patients with and without ADHD (*N* = 804)

	Index suicide attempt^**a**^					Index NSSI				
	ADHD, ***n*** = 66	No ADHD, ***n*** = 600	***χ***^***2***^	***df***	***P***	ADHD, ***n*** = 27	No ADHD, ***n*** = 111	***χ***^***2***^	***df***	***P***
	n (%)	n (%)				n (%)	n (%)			
**Method**^**b**^										
Poisoning^c^	39 (59)	361 (60)	0.03	1	.87, ns	7 (26)	19 (17)	1.10	1	.29, ns
Gassing	1 (2)	5 (0.8)	0.31	1	.58, ns	–	–	–	–	–
Hanging/strangulation/choking	8 (12)	70 (12)	0.01	1	.91, ns	2 (7)	3 (3)	1.38	1	.24, ns
Drowning	–	15 (3)	1.69	1	.19, ns	–	–	–	–	–
Cutting	12 (18)	105 (18)	0.02	1	.89, ns	16 (59)	79 (71)	1.44	1	.23, ns
Jumping	2 (3)	21 (4)	0.04	1	.84, ns	–	–	–	–	–
Vehicular	3 (5)	23 (4)	0.08	1	.78, ns	–	1 (0.9)	0.25	1	.62, ns
Firearm/explosives	–	3 (0.5)	0.33	1	.57, ns	–	–	–	–	–
Other method	3 (5)	15 (3)	0.95	1	.33, ns	6 (22)	15 (14)	1.28	1	.26, ns

*ADHD* Attention deficit hyperactivity disorder, *NSSI* Non-suicidal self-injury

^a^Only actual suicide attempts as defined by the Columbia Suicide Severity Rating Scale

^b^More than one method could be reported

^c^Single method

For those with an actual attempt, self-poisoning was employed as a single method in 400 persons (60%); proportions were nearly identical in patients with and without ADHD.

NSSI at baseline was more common among those with ADHD (Table 2). No gender difference was observed in terms of the prevalence of ADHD (70 out of 541 women, 13%, and 23 out of 263 men, 9%, *P =* .08). Median age was lower (*U* = 21,322, *z* = − 5.58, *P* < .001) in persons with ADHD (Mdn = 29) than in those without (Mdn = 39). Participants with ADHD were less likely to have a clinical diagnosis of depression at discharge compared to those without ADHD, but proportions with anxiety disorder were similar. A clinical diagnosis of alcohol use disorder was somewhat less common in the ADHD group but the difference in proportions did not reach significance. Just over one tenth of the participants in both groups had a drug misuse disorder. Almost a third of those with ADHD had a clinical diagnosis of personality disorder, compared to one fifth of those without ADHD. Emotionally unstable personality disorder (EUPD) specifically was not more prevalent among persons with ADHD (24% vs 16%, *P =* 0.07). Autism spectrum disorders were recorded in over one fifth of the participants in the ADHD group.

**Table 2:** Baseline sociodemographic and clinical characteristics of participants with and without ADHD (*N* = 804)

	ADHD, ***n*** = 93	No ADHD, ***n*** = 711	***χ***^***2***^	***df***	***P***	Missing data
	n (%)	n (%)				n^**a**^
**Non-suicidal self-injury (NSSI)**	27 (29)	111 (16)	10.4	1	**.001**	–
**Women**	70 (75)	471 (66)	3.04	1	.08, ns	–
**Age group**						
18–44	81 (87)	461 (65)	18.55	1	**<.001**	–
45–64	11 (12)	169 (24)	6.75	1	**.01**	–
65+	1 (1)	81 (11)	9.56	1	**.002**	–
**Clinical diagnosis**^**b**^						
Alcohol use disorder (F10.1/F10.2)	4 (4)	76 (11)	3.75	1	.05, ns	–
Drug misuse disorder (F11-F19, F17 excluded)	12 (13)	78 (11)	0.31	1	.58, ns	–
Depression (F32.X and/or F33.X)	12 (13)	218 (31)	12.7	1	**<.001**	–
Anxiety, stress (F40–49)	42 (45)	278 (39)	1.26	1	.26, ns	–
Personality disorder (F60–69)	28 (30)	142 (20)	5.07	1	**.02**	–
Emotionally unstable personality disorder (F60.3)	22 (24)	115 (16)	3.26	1	0.07, ns	–
Autism spectrum disorder (F80–89)	21 (23)	33 (5)	42.2	1	**<.001**	–
WHO hazardous use cut-off, AUDIT	34 (51)	256 (48)	0.24	1	.63, ns	199
Trait impulsivity, SUAS 11	58 (62)	283 (40)	17.1	1	**<.001**	–
Impulsivity at index suicide attempt (*n* = 666), SIS 15	39 (60)	251 (43)	7.04	1	**.01**	14
High medical lethality, index suicide attempt (n = 666), C-SSRS	16 (25)	196 (35)	2.39	1	.12, ns	36
Recent relationship problems	70 (75)	456 (64)	4.44	1	**.04**	1

*ADHD* Attention deficit hyperactivity disorder, *AUDIT* Alcohol Use Disorders Identification Test, *SUAS 11* Suicide Assessment Scale item 11, *SIS 15* Suicide Intent Scale item 15, *C-SSRS* Columbia Suicide Severity Rating Scale

^a^No significant differences in proportions missing between groups

^b^Participants could have more than one diagnosis

Table 2 shows further that the impulsivity trait was significantly more common among persons with ADHD, as was impulsivity in connection with the index episode in those with actual attempts. We found no difference in proportions with high medical lethality between groups in attempters with and without ADHD. Applying a higher cut-off of 4 points on the C-SSRS lethality item did not change results (results not shown).

AUDIT data were available for 605 persons. Almost half of these (48%) had AUDIT scores above the WHO cut-off for hazardous use. Similar proportions of hazardous use were seen in participants with and without ADHD (Table 2). For those with data on the MADRS-S, mean scores were significantly higher among persons with ADHD (31.7 vs 27.2, *P* < .001). Among persons with a clinical diagnosis of depression, mean MADRS-S scores were higher in those with ADHD compared to those without (38.6 vs 27.6, *P =* .001). Anxiety scores were also higher in the ADHD group (self-rated BSA: 24.8 vs 21.5, *P =* .004). Three quarters of those with ADHD reported having experienced recent relationship problems prior to index episode, a proportion higher than that observed in participants without ADHD.

### Six-month follow-up

While proportions with psychiatric outpatient care after the index episode were similar between groups, patients with ADHD were more likely to have at least one subsequent consultation with psychiatric emergency services within the 6-month observation period (Table 3). Participants with ADHD were less likely to have an antidepressant prescription during aftercare. Almost half of those in the ADHD group were prescribed antipsychotics during follow-up, a proportion greater than that in participants without ADHD.

**Table 3:** Distribution of follow-up care during 6 months after index episode (suicide attempt or non-suicidal self-injury)

	ADHD, ***n*** = 93 n (%)	No ADHD, ***n*** = 711 n (%)	***χ***^***2***^	***df***	***P***	Missing data n^a^
**Type of care**						
Recorded visit to psychiatric outpatient care	78 (84)	537 (77)	2.48	1	.12, ns	10
Recorded visit psychiatric emergency services	51 (55)	270 (38)	9.28	1	.002	7
Recorded visit to treatment for substance abuse	20 (22)	124 (18)	0.81	1	.37, ns	10
Referral to primary care	11 (12)	143 (20)	3.73	1	.05, ns	4
Psychological treatment	48 (52)	301 (43)	2.77	1	.10, ns	3
**Pharmacological treatment**						
Antidepressants	56 (60)	526 (75)	9.37	1	.002	11
Mood stabilizers	17 (19)	119 (17)	0.10	1	.76, ns	19
Antipsychotics	45 (49)	258 (37)	4.77	1	.03	17
Anxiolytics	80 (87)	586 (84)	0.47	1	.49, ns	16
ADHD medication	67 (73)	–	**–**	**–**	**–**	17

*ADHD* Attention deficit hyperactivity disorder

^a^No significant differences in proportions missing between groups

At least one suicide attempt or suicide death was recorded during the 6-month observation period in 29% of those with ADHD (27 out of 93). The corresponding proportion in the group without ADHD was 19% (*n* = 138, *χ*^*2*^ = 4.67, *df* = 1, *P =* .03). Proportions with suicidal behaviour during follow-up in each group were only slightly attenuated after exclusion of participants with EUPD (ADHD 25%, no ADHD 16%, *χ*^*2*^ = 3.83, *df* = 1, *P =* .050). Methods employed in connection with actual suicide attempts during the follow-up period are shown by ADHD status in Table 4. Hanging/strangulation was employed by 30% of the ADHD group, and only 13% of the others (*P =* .03). Two of the episodes among persons with ADHD were fatal, as were 8 of those in the group without ADHD (*P =* .41, ns).

**Table 4:** Methods employed in suicidal behaviour during 6-month follow-up. Includes both fatal and non-fatal events

	ADHD, ***n*** = 27 n (%)	No ADHD, ***n*** = 138 n (%)	***χ***^***2***^	***df***	***P***
**Method**^**a**^					
Poisoning	18 (67)	108 (78)	1.68	1	.20, ns
Gassing	–	–	–	–	–
Hanging/strangulation/choking	8 (30)	18 (13)	4.68	1	.03
Drowning	–	2 (1)	0.40	1	.53, ns
Cutting	3 (11)	24 (17)	0.65	1	.42, ns
Jumping	–	–	–	–	–
Vehicular	1 (4)	2 (1)	0.64	1	.42, ns
Firearm/explosives	–	–	–	–	–
Other method	1 (4)	6 (4)	0.023	1	.88, ns

*ADHD* Attention deficit hyperactivity disorder.

^a^Only one method was registered for each event at follow-up

All logistic regression models for the whole sample are detailed in Table 5. A binary logistic regression model revealed a 70% increase in odds for fatal or non-fatal suicidal behaviour during the 6-month follow-up period among participants with ADHD (crude OR = 1.70, 95% CI [1.05, 2.76], Wald χ^2^(1) = 4.59, *P =* .03) compared to those without. Risk of at least one fatal/non-fatal suicidal behaviour remained elevated for the ADHD group also after adjustment for clinical diagnosis of depression at baseline (OR = 1.65, 95% CI [1.01–2.68], Wald χ^2^(1) = 3.97, *P =* .046). Adding EUPD to the model lowered impact of ADHD below the significance threshold, although not by much (OR = 1.58, 95% CI [0.96–2.60], Wald χ^2^(1) = 3.22, *P =* .073). The predictive OR for EUPD was high in this model (OR = 2.80, 95% CI [1.86–4.22], Wald χ^2^(1) = 24.30, *P =* <.001). Table 5 shows further that ADHD was not associated with suicidal behaviour during follow-up in the final regression model but the association between EUPD and new SA was only slightly attenuated after inclusion of sex, age and trait impulsivity. In this final model, age was an independent predictor of new SA (OR = 0.98, 95% CI [0.97–0.99], Wald χ^2^(1) = 10.54, *P =* .001), but neither sex nor impulsivity were significant predictors. Stratified regression models on the low and middle age groups (18–44, 45–64, 65+ not tested because only one participant had ADHD) did not yield significant ORs for ADHD. Stratified regression models are detailed in Supplementary Table S1.

**Table 5:** Logistic regression models, prediction of new suicide attempts (fatal and non-fatal) within 6 months

	Model			Predictor						
	Nagelkerke ***R***^***2***^	***χ***^***2***^	***P***	B	SE	Wald	***df***	***P***	Exp(B)	95% CI
ADHD	.008	4.33	.04							
ADHD				0.53	0.25	4.59	1	.032	1.70	1.05–2.76
ADHD + depression	.010	5.24	.07*, ns*							
ADHD				0.50	0.25	3.97	1	.046	1.65	1.01–2.68
Depression				−0.19	0.20	0.89	1	.35, ns	0.83	0.56–1.23
ADHD + depression + EUPD	.054	28.37	<.001							
ADHD				0.46	0.26	3.22	1	.073, ns	1.58	0.96–2.60
Depression				−0.28	0.21	0.02	1	.893, ns	0.97	0.65–1.46
EUPD				1.03	0.21	24.30	1	< 0.001	2.80	1.86–4.22
ADHD + depression + EUPD + trait impulsivity (SUAS 11) + sex + age	.072	37.67	<.001							
ADHD				0.34	0.26	1.67	1	.20, ns	1.40	0.84–2.32
Depression				0.08	0.21	0.15	1	.70, ns	1.09	0.72–1.65
EUPD				1.00	0.22	20.11	1	< 0.001	2.69	1.75–4.14
Trait impulsivity (SUAS 11)				0.14	0.19	0.53	1	.47, ns	1.15	0.79–1.68
Sex				−0.22	0.20	1.22	1	.27, ns	0.80	0.54–1.19
Age				−0.02	0.01	5.87	1	.015	0.99	0.97–1.00

*ADHD* Attention deficit hyperactivity disorder, *EUPD* Emotionally unstable personality disorder, *SUAS* Suicide Assessment Scale item 11, 2 points or more

## Discussion

In this prospective study of adults who sought hospital care in connection with self-harm, we found that ADHD was associated with a particularly elevated risk of subsequent suicidal behaviour. Patients with hyperactivity disorders were less likely to have received a clinical diagnosis of depression and more likely to have a personality disorder. While the index suicide attempts of those with ADHD were more often characterized as impulsive, proportions with high medical lethality did not differ in suicide attempters with and without ADHD. In the total group, relationship problems were more prevalent in persons with ADHD.

While a relatively small proportion (13%) of the patients with ADHD received a clinical diagnosis of depression in connection with the index self-harm episode, self-rated MADRS scores were higher in those with ADHD, both in the total group and in those with a clinical diagnosis of depression. This might represent an actual between-group difference in affective psychopathology, but another possibility might be that persons with ADHD perceive and self-rate the MADRS items differently than their peers without ADHD. It should be kept in mind that one quarter of the patients with ADHD also had a clinical diagnosis of autism spectrum disorder, which might impact on the manner in which they respond to the MADRS items [26]. While there was no significant difference in proportions with EUPD between groups, our regression analyses showed that EUPD has a large impact on repeat suicidal behaviour regardless of ADHD status. Phenotypic similarities between ADHD and EUPD make interpretation of these results difficult, and further research is warranted to assess specific differences in symptoms between these groups in the context of suicidal behaviour.

Neither alcohol use nor drug misuse disorders were more prevalent among persons with ADHD. Further, AUDIT scores and proportions scoring above the WHO cut-off for hazardous alcohol use were similar between groups. Taken together, these findings were somewhat unexpected as previous studies involving both clinical [27] and prison [28] settings have reported larger proportions with drug misuse disorders among persons with ADHD than in those without. However, it must be stressed that participants in our study were recruited on the basis on self-harm behaviour and the quality of evidence for an association between alcohol and drug misuse and suicide is strong [29].

One explanation for the increased risk for fatal/non-fatal suicidal behaviour during follow-up in those with ADHD might be the greater degree of impulsivity in patients with this disorder. For those 66 persons with ADHD whose index episodes were actual suicide attempts, these were characterized by impulsivity to a greater degree than in those without ADHD. It has been suggested that trait impulsivity might provide an important mechanism behind the shared genetic risk observed in ADHD and suicide [15]. In our present study, patients with ADHD scored higher on the impulsivity item of the SUAS, but our fully adjusted regression model did not show significantly increased risk of new SA if a person displayed the impulsivity trait.

As far as we are aware, this is to date the largest prospective clinical study examining the relationship between ADHD and risk for future suicidal behaviour in adults who self-harm. Participants were well-characterized at the index episode, enabling comparisons of clinical characteristics between persons with and without ADHD. Using ADHD medication as a diagnostic proxy should improve diagnostic validity as these drugs are licensed in Sweden and may only be prescribed on strict indication by board-certified psychiatrists. Comorbidity was examined not only by clinical diagnosis but also by self-rating scales, yielding dimensional measures of affective symptomatology and problematic alcohol use. Trained mental health professionals carried out the interviews, which allowed us to expand on findings of previous register-based Swedish research by providing individual-level data on impulsivity, medical lethality, and recent relationship problems. The participation rate for this study was high, exceeding in total 70%. Both the wider age range (including also middle-aged patients with ADHD) and the shorter follow-up time increase relevance of our findings for adult psychiatric services. The short follow-up interval is clinically relevant in an assessment of a clinical sample [30], especially considering the particularly high risk of suicide in patients with recent psychiatric hospitalization [31]. While the number of patients included in this study is relatively large compared to many other prospective clinical studies on self-harm, the size of the ADHD group is not, which limited study power. As expected, ages were skewed towards younger in the ADHD group which makes interpretation of the final regression model difficult. The odds ratio for ADHD remained high in this final model which may suggest significance was lost as a result of low power and overlapping variability with age.

In accordance with our ethical permission and the European General Data Protection Regulation law (GDPR), we were not allowed to collect any data on those who declined participation. Thus, comparisons between non-participants and participants were not possible and reasons for non-participation were not registered. We relied on clinical data to identify persons with ADHD, and a limitation is thus that we lacked a valid instrument for the evaluation of hyperactivity symptoms. Further, we had no objective measures of cognitive functioning and problem-solving style, which might differentiate patients with and without ADHD. Another consideration is that we did not have access to data on childhood and adolescent psychiatric history (self-harm, conduct disorders, school achievement, etc) which might contribute differentially to the suicidal process in participants with and without ADHD. We did not have access to information on specific types of psychological treatments administered after the index episode, and various forms of treatment could have had differential effects on new suicide attempts during follow-up. Prospective studies in diverse settings are called for to further characterize the clinical features and subsequent risk of self-harm behaviour in adults with ADHD.

## Conclusions

ADHD was prospectively associated with suicidal behaviour, although this association was no longer statistically significant when accounting for age and EUPD. Several of our findings might inform clinicians who evaluate and treat patients with ADHD and self-harm issues. As the proportion with high lethality attempts was just as large in those with ADHD as in those without ADHD, this highlights the need for careful evaluation in the clinical context. The finding that persons with ADHD were more likely to use hanging or strangulation during follow-up compared to those without ADHD suggests a tendency towards more violent behaviour. Taken together, our findings highlight that the higher degree of impulsivity in the attempts of persons with ADHD must not be mistakenly equated with low degree of suicide risk. Impulsive attempts are just as likely to result in medical injury as more planned attempts [32]. Another finding of clinical relevance was that three-quarters of the patients with ADHD reported recent relationship issues. The quality of evidence for the association between recent life events and suicide is high [29], and our findings suggest that elevated exposure to relationship problems in the ADHD group might add to the suicidal diathesis. More research is needed to determine whether this is mediated by co-morbid EUPD. Results can provide clues for targeted interventions to reduce suicide risk in persons with ADHD.

## Supplementary Information

**Additional file 1: Supplementary Table S1.** Logistic regression models stratified by age groups, prediction of new suicide attempts (fatal and non-fatal) within 6 months.

## Data Availability

In accordance with our ethics board approval, data generated for the current study are not publicly available for ethical reasons. However, group level data are available from the corresponding author on reasonable request. Further, we are willing to make an application to the national ethics board to ask if they would consider authorising data sharing of individual level data, should there arise the need for individual level data to be made available.

## Abbreviations

ADHD: Attention deficit and hyperactivity disorder
SA: Suicide attempt
NSSI: Non-suicidal self-injury
C-SSRS: Columbia Suicide Severity Rating Scale
AUDIT: Alcohol Use Disorders Identification Test
SUAS: Suicide Assessment Scale
SIS: Suicide Intent Scale
MADRS: Montgomery-Åsberg Depression Rating Scale
BSA: Brief Scale for Anxiety
ICD-10: 10th revision of the International Statistical Classification of Diseases and Related Health Problems
ADD: Attention deficit disorder
DAMP: Dysfunction of attention, motor control and perception
MVA: Missing value analysis
MCAR: Missing completely at random
EM: Expected maximum
MAR: Missing at random
EUPD: Emotionally unstable personality disorder
GDPR: General Data Regulation Protection
