# Supplementary material for: Attention deficit hyperactivity disorder in adults who present with self-harm: a comparative 6-month follow-up study
Source: BMC Psychiatry. 2022 Jun 24;22:428. doi: 10.1186/s12888-022-04057-0 (PMC9233312; doi:10.1186/s12888-022-04057-0)
Supplement: Supplementary file 1 — Additional file 1: Supplementary Table S1. Logistic regression models stratified by age groups, prediction of new suicide attempts (fatal and non-fatal) within 6 months. [file 12888_2022_4057_MOESM1_ESM.docx]

| **Supplementary Table S1.** Logistic regression models stratified by age groups, prediction of new suicide attempts (fatal and non-fatal) within 6 months. | | | | | | | | | | | | |
| --- | --- | --- | --- | --- | --- | --- | --- | --- | --- | --- | --- | --- |
|  | **Model** | | |  | **Predictor** | | | | | | | |
|  | **Nagelkerke *R^2^*** | ***χ^2^*** | ***P*** |  | | **B** | **SE** | **Wald** | ***df*** | ***P*** | **Exp(B)** | **95% CI** |
|  |  |  |  |  | |  |  |  |  |  |  |  |
| ADHD + depression + EUPD + sex (ages 18-44) | .058 | 21.11 | <.001 |  | |  |  |  |  |  |  |  |
| ADHD |  |  |  |  | | -0.43 | 0.28 | 2.43 | 1 | .12, ns | 1.54 | 0.89-2.66 |
| Depression |  |  |  |  | | 0.26 | 0.25 | 1.09 | 1 | .30, ns | 1.30 | 0.80-2.12 |
| EUPD |  |  |  |  | | 1.06 | 0.24 | 19.16 | 1 | <.001 | 2.89 | 1.80-4.64 |
| Sex |  |  |  |  | | -0.27 | 0.24 | 1.26 | 1 | .26, ns | 0.77 | 0.48-1.22 |
| ADHD + depression + EUPD (ages 45-64) | .060 | 6.49 | .17, *ns* |  | |  |  |  |  |  |  |  |
| ADHD |  |  |  |  | | 0.59 | 0.73 | 0.65 | 1 | .42, ns | 1.80 | 0.43-7.59 |
| Depression |  |  |  |  | | -0.41 | 0.50 | 0.68 | 1 | .41, ns | 0.66 | 0.25-1.77 |
| EUPD |  |  |  |  | | 1.20 | 0.56 | 4.57 | 1 | .03 | 3.33 | 1.11-10.05 |
| Sex |  |  |  |  | | -0.13 | 0.45 | 0.08 | 1 | 0.78, ns | 0.88 | 0.37-2.11 |

ADHD: Attention Deficit Hyperactivity Disorder, EUPD: Emotionally Unstable Personality Disorder, SUAS 11: Trait impulsivity, Suicide Assessment Scale item 11, 2 points or more.
